# Evaluation of the risk of acute kidney injury with the use of piperacillin/tazobactam among adult critically ill patients

**DOI:** 10.1007/s15010-020-01480-x

**Published:** 2020-07-22

**Authors:** Mohamed O. Saad, Adham M. Mohamed, Hassan A. Mitwally, Ahmed A. Shible, Ali Ait Hssain, Mohamed A. Abdelaty

**Affiliations:** 1 Pharmacy Department, Hamad Medical Corporation, Doha, Qatar; 2 Pharmacy Department, Saint Luke’s Hospital of Kansas City, 4401 Wornall Road, Kansas City, MO 64111 USA; 3 Medical Intensive Care Unit, Hamad Medical Corporation, Doha, Qatar

**Keywords:** Acute kidney injury, Adverse event, Critically ill, Piperacillin/tazobactam, Renal, Nephrotoxicity

## Abstract

**Purpose:**

Piperacillin/tazobactam (PT), when combined with vancomycin, is associated with an increased risk of acute kidney injury (AKI). It is not known whether PT alone is associated with a higher incidence of AKI compared to other β-lactams among critically ill patients. The objective of this study was to compare the incidence of AKI associated with the use of PT to other β-lactams among adult critically ill patients

**Methods:**

This retrospective study was conducted in the surgical and the medical intensive care units at two hospitals within Hamad Medical Corporation (HMC) in Qatar and included adult critically ill patients who received at least one dose of anti-pseudomonal β-lactams. The primary outcome was acute kidney injury, defined using the Kidney Disease Improving Global Outcomes (KDIGO) criteria. Multiple logistic regression with adjustment for pre-specified potential confounders was used for the primary outcome analysis.

**Results:**

A total of 669 patients were included in the analysis: 507 patients in the PT group and 162 patients in the control (meropenem/cefepime) group. AKI occurred in 136 (26.8%) members of the PT group and 38 (23.5%) members of the control group [odds ratio (OR) 1.2; 95% confidence interval (CI) 0.79–1.8]. The results were not significantly altered after adjusting for the pre-specified potential confounders (adjusted OR 1.38; 95% CI 0.88–2.15).

**Conclusion:**

In this study, PT was not associated with a higher risk of AKI compared to cefepime or meropenem among adult critically ill patients.

**Electronic supplementary material:**

The online version of this article (10.1007/s15010-020-01480-x) contains supplementary material, which is available to authorized users.

## Introduction

β-Lactam antibiotics are commonly prescribed in the intensive care unit (ICU) to treat confirmed or suspected bacterial infections [1]. These antibiotics have a wide spectrum of coverage with a highly acceptable safety profile, making them the drug of choice for many bacterial infections (e.g., pneumonia, urinary tract infection, meningitis, and intra-abdominal infection). Piperacillin/tazobactam (PT), a combination of a penicillin (piperacillin) and a β-lactamase inhibitor (tazobactam), is one of the most commonly prescribed anti-pseudomonal β-lactam antibiotics [2]. In fact, according to a 2011 survey of the prevalence of antimicrobial medications in acute care hospitals in the United States, PT was the second most common antibiotic used to treat infections in critically ill patients, comprising 25.6% of all antimicrobial medications given to critically ill patients, and it was among the top five antibiotics given to non-critically ill patients [3]. It exhibits antibacterial activity against Gram-positive, Gram-negative, and anaerobic pathogens.

Acute kidney injury (AKI) affects approximately 55–60% of critically ill patients [4, 5]. Both the incidence and the severity of AKI are associated with an increased risk of mortality in this population [4–6]. Therefore, it is important to avoid drugs associated with AKI, whenever possible, to decrease the risk of mortality among critically ill patients.

Recently, several studies found an association between the combination of PT plus vancomycin and acute kidney injury (AKI) compared to the combination of other β-lactams (e.g., cefepime or meropenem) plus vancomycin [7–10]. These studies found that the combination of PT and vancomycin is an independent risk factor for AKI after adjustment for potential confounders. Later, several meta-analyses confirmed the increased risk of AKI when PT was combined with vancomycin in both adult and pediatric populations [11–15].

Because vancomycin was a common factor when comparing PT plus vancomycin to other β-lactams plus vancomycin, it appears that vancomycin is not the cause of increased nephrotoxicity. Additionally, in some of these studies, the risk of AKI was higher with the combination of PT plus vancomycin compared to vancomycin alone [12, 14, 15]. A finding which raises a concern about the renal safety of PT monotherapy.

Although few studies examined the effect of PT monotherapy on renal function, these studies either did not directly assess this association among adult critically ill patients [16–18] or had major limitations that render their conclusions about renal safety of PT questionable [19].

Due to the need to explore the cause of increased AKI with the combination of PT and vancomycin and the paucity of data about the incidence of nephrotoxicity with PT alone, the renal safety of PT monotherapy among critically ill patients has yet to be determined.

## Aim of the study

This study was conducted to examine the incidence of acute kidney injury with the use of PT, without vancomycin, among adult critically ill patients compared to other β-lactams.

## Methods

This was a retrospective observational study intended to assess differences in the incidence of AKI between adult critically ill patients receiving PT and those receiving cefepime or meropenem (the control group) without concomitant use of vancomycin. This study was conducted in the medical and surgical intensive care units at two hospitals within Hamad Medical Corporation (HMC) in Qatar: Hamad General Hospital and Al-Wakra Hospital. The study was approved by the HMC Medical Research Center (research number: MRC-01-17-043). We included adult (≥ 18 years old) patients admitted to medical or surgical intensive care units between June 1st, 2015, and April 30th, 2018, who received at least one dose of any of the three medications (PT, meropenem, or cefepime). Patients were excluded if they had baseline creatinine clearance (CrCl) below 30 mL/min, were on renal replacement therapy before initiation of the index drug, received vancomycin concomitantly, or were shifted from one study drug to another.

After obtaining approval from the institutional review board, the data of the eligible patients were collected from electronic medical records. We collected the following data: type of critical care unit (medical or surgical); age; sex; weight; pregnancy status (for females); drug allergies; diagnosis at ICU admission; source of infection; duration of therapy with the index drug; baseline serum creatinine (most recent value before administration of index drug); baseline Acute Physiology and Chronic Health Evaluation (APACHE) II score; vasopressor use; invasive mechanical ventilation; history of heart failure, liver cirrhosis, chronic kidney disease, hypertension, or diabetes mellitus; and concurrent use of nephrotoxic drugs [amikacin, gentamicin, colistin, vancomycin, teicoplanin, amphotericin, furosemide, angiotensin-receptor blocker (ARB), angiotensin-converting-enzyme inhibitor (ACEI), tacrolimus, or cyclosporine].

The primary outcome was acute kidney injury defined using the Kidney Disease Improving Global Outcomes (KDIGO) criteria as an increase in serum creatinine by 26.5 µmol/L (0.3 mg/dL) or more within 48 h or an increase by 50% or more from baseline within 7 days. Serum creatinine values up to 3 days after completion of therapy were included in the analysis. The secondary outcomes were renal replacement therapy, time to AKI, mortality, hospital length of stay, and length of stay in the ICU. Patients’ baseline characteristics and outcome data were described as means with the standard deviation for continuous variables, medians with the interquartile range for ordinal variables, and frequencies with percentages for categorical variables. We compared the baseline characteristics using the *t* test for normal continuous variables, the Wilcoxon rank-sum test for ordinal and non-normal continuous variables, and the Chi-square test for categorical variables.

Multiple logistic regression adjusted for pre-specified potential confounders; including age, baseline CrCl, hypertension, diabetes, heart failure, liver cirrhosis, concomitant vasopressor therapy, duration of therapy, and the number of concomitant nephrotoxic medications; was used for the primary outcome analysis. Two additional analyses were conducted to confirm the results of the primary analysis; the first was a multiple logistic regression model that included the baseline imbalances (*P* < 0.1) in addition to the pre-specified confounders and the second was the latter regression model after excluding patients who received teicoplanin concomitantly. All *P* values were two sided, and results with *P* values of less than 0.05 were considered significant. Statistical analyses were conducted using the IBM Statistical Package for the Social Sciences (SPSS) version 22 (IBM Corp., Armonk, NY).

## Results

A total of 669 patients were included in the analysis: 507 patients in the PT group and 162 patients in the meropenem/cefepime (control) group. About two-third of the patients were male, and the average age was 51.7 years. The median APACHE II score was 10, and one-third of the patients were postoperative. Before receiving the index β-lactam therapy, 21.7% of patients were on vasopressor therapy and 29.3% were on mechanical ventilation support. The baseline characteristics of the groups were comparable. However, the PT group had more postoperative patients and higher CrCl, as shown in Table 1. Besides, the mean duration of therapy with the index β-lactam was shorter in the PT group than in the control group (5.9 ± 4.3 vs. 6.9 ± 5.1 days; *P* = 0.01). Concomitant treatments were comparable between the two groups, except for calcineurin inhibitors, colistin, and teicoplanin, which were more frequently used in the control group (Table 2). Sources of infection are described in Table 1 of the supplementary appendix.

**Table 1:** Baseline characteristics of patients

Characteristics	PT (*n* = 507)	Control (*n* = 162)	*P* value	All patients (*n* = 669)
Male sex, *n* (%)	326 (64.3)	100 (61.7)	0.55	426 (63.7)
Age [years], mean (SD)	51.1 (18.4)	53.4 (17.4)	0.17	51.7 (18.2)
Weight [kg], mean (SD)	76.6 (21.2)	73 (18.3)	0.05	75.7 (20.6)
Hypertension, *n* (%)	201 (39.6)	72 (44.4)	0.28	273 (40.8)
Diabetes, *n* (%)	179 (35.3)	65 (40.1)	0.27	244 (36.5)
Chronic liver disease, *n* (%)	25 (4.9)	11 (6.8)	0.36	36 (5.4)
Heart failure, *n* (%)	68 (13.4)	23 (14.2)	0.80	91 (13.6)
Chronic lung disease, *n* (%)	60 (11.8)	27 (16.7)	0.11	87 (13)
Pregnant, *n* (%)	7 (1.4)	5 (3.1)	0.18	12 (1.8)
Post-operative, *n* (%)	200 (39.4)	36 (22.2)	< 0.001	236 (35.3)
APACHE II score, median (IQR)	10 (10)	11 (8.25)	0.37	10 (9)
Serum creatinine [µmol/L], mean (SD)	88.5 (44.8)	93.5 (47.2)	0.22	89.7 (45.4)
CrCl [mL/min], mean (SD)^a^	106.8 (59.7)	93.6 (45.3)	0.01	103.6 (56.8)
Vasopressor therapy prior to beta-lactam therapy, *n* (%)	105 (20.7)	40 (24.7)	0.28	145 (21.7)
Mechanical ventilation, *n* (%)	147 (29)	49 (30.2)	0.76	196 (29.3)

*APACHE II* Acute Physiology and Chronic Health Evaluation II, *CrCl* creatinine clearance, *IQR* interquartile range, *PT* piperacillin/tazobactam, *SD* standard deviation

^a^Creatinine clearance was estimated using Cockroft–Gault formula

**Table 2:** Concomitant therapeutic interventions

Characteristics	PT (*n* = 507)	Control (*n* = 162)	*P* value	All patients
Vasopressor therapy, *n* (%)	171 (33.7)	54 (33.3)	0.93	225 (33.6)
Mechanical ventilation, *n* (%)	203 (40)	57 (35.2)	0.27	260 (38.9)
Number of nephrotoxic medications, median (IQR)	1 (1)	1 (1)	0.25	1 (1)
ACEI, *n* (%)	59 (11.6)	19 (11.7)	0.98	78 (11.7)
Aminoglycoside, *n* (%)	10 (2)	7 (4.3)	0.15	17 (2.5)
Amphotericin, *n* (%)	3 (0.6)	2 (1.2)	0.60	5 (0.7)
Calcineurin inhibitor, *n* (%)	11 (2.2)	9 (5.6)	0.04	20 (3)
Colistin, *n* (%)	1 (0.2)	4 (2.5)	0.01	5 (0.7)
Teicoplanin, *n* (%)	7 (1.4)	9 (5.6)	0.01	16 (2.4)
Loop diuretics, *n* (%)	237 (46.7)	70 (43.2)	0.43	307 (45.9)

*ACEI* angiotensin-converting enzyme inhibitors, *IQR* interquartile range, *PT* piperacillin/tazobactam

AKI occurred in 26% (174 patients) of the study population; with no statistically significant difference between the two groups [136 patients (26.8%) in the PT group vs. 38 patients (23.5%) in the control group; odds ratio (OR) 1.2, 95% confidence interval (CI) 0.79–1.8; *P* = 0.395]. After including the pre-specified potential confounders (age, baseline CrCl, hypertension, diabetes, heart failure, liver cirrhosis, concomitant vasopressor therapy, duration of therapy, and the number of concomitant nephrotoxic medications) in the regression model, the adjusted odds ratio was 1.38 and the 95% CI was 0.88–2.15. The difference remained non-significant after adjusting for the baseline imbalances (weight, post-operative status, concomitant use of calcineurin inhibitors, concomitant use of colistin, and concomitant use of teicoplanin) in the regression model (adjusted OR 1.58, 95% CI 0.99–2.52). However, patients who received teicoplanin concomitantly were excluded, the incidence of AKI was significantly higher in the PT group (*n* = 653; adjusted OR 1.68, 95% CI 1.04–2.72). Among the secondary renal endpoints, the median time to AKI was significantly shorter in the PT group (0.94 days vs. 2.08 days; *P* = 0.009) while the need for renal replacement therapy was significantly higher in the control group (2.2% in the PT group vs. 5.6% in the control group; *P* = 0.03) (Table 3). Regarding other clinical outcomes, mortality did not differ between the groups, but the length of hospital and ICU stay were higher in the control group (Table 4).

**Table 3:** Renal safety outcomes of patients

Primary outcome	PT (*n* = 507)	Control (*n* = 162)	Crude OR (95% CI)	Adjusted OR^a^ (95% CI)
Acute kidney injury, *n* (%)	136 (26.8)	38 (23.5)	1.2 (0.79–1.8)	1.38 (0.88–2.15)

Secondary outcomes	PT (*n* = 507)	Control (*n* = 162)	*P* value
Need for renal replacement therapy, *n* (%)	11 (2.2)	9 (5.6)	0.04
Time to acute kidney injury [days], median (IQR)^b^	0.94 (2.02)	2.08 (6.13)	0.009
Time to renal replacement therapy [days], median (IQR)^b^	4.4 (8.2)	1.7 (1.9)	0.21

Acute kidney injury was defined using the Kidney Disease Improving Global Outcomes (KDIGO) criteria as an increase in serum creatinine by 26.5 µmol/L (0.3 mg/dL) or more within 48 h or an increase by 50% or more from baseline within 7 days

*PT* piperacillin/tazobactam, *OR* odds ratio, *IQR* interquartile range

^a^Adjusted for age, baseline creatinine clearance, hypertension, diabetes, heart failure, liver cirrhosis, concomitant vasopressor therapy, duration of therapy and number of concomitant nephrotoxic medications

^b^Calculated only among patients who had the outcome

**Table 4:** Other outcomes of patients

Outcome	PT (*n* = 507)	Control (*n* = 162)	*P* value
Mortality, *n* (%)	61 (12)	19 (11.7)	0.92
Length of ICU stay [days], median (IQR)^a^	3.7 (5)	4.9 (7)	0.03
Length of hospital stay [days], median (IQR)^a^	12.8 (18.2)	18.2 (28)	< 0.001

*ICU* intensive care unit, *IQR* interquartile range, *PT* piperacillin/tazobactam

^a^Calculated among survivors only. ICU stay was calculated as sum of all ICU days during the index hospitalization

## Discussion

The purpose of this study was to evaluate the incidence of AKI when critically ill patients were administered PT compared to cefepime or meropenem (control group). In this study, there was no statistically significant difference in the primary endpoint of AKI between the PT group and the control group.

Several studies have shown that combining PT and vancomycin leads to an increased risk of AKI, compared to the combinations of other β-lactams with vancomycin [7–10]. Multiple meta-analyses have confirmed this association among adult and pediatric patients [11–15]. This finding has been specifically demonstrated among critically ill patients who have a higher baseline risk of AKI [10, 12].

The mechanism of increased nephrotoxicity with this combination is still not well understood. It might be thought that the increased risk is due to the nephrotoxicity of vancomycin. However, this assumption is not supported because vancomycin was a common factor when comparing PT plus vancomycin to other β-lactams plus vancomycin and the risk of AKI was higher with the combination of PT plus vancomycin compared to vancomycin alone [12, 14, 15]. There are two other possible explanations: either PT has an independent nephrotoxic effect or it has a synergistic effect on vancomycin-associated nephrotoxicity. Piperacillin has been shown to have a higher affinity for the renal organic anion transporter (OAT) compared to another penicillin drug, flucloxacillin [20]. Additionally, a previous case report identified interstitial nephritis as a mechanism of PT-associated AKI [21]. However, PT has not been previously considered a nephrotoxic drug [12].

Limited studies assessed the effect of PT monotherapy on renal function. Karino et al. compared PT to biapenem for the treatment of healthcare-associated pneumonia among elderly patients, observing nephrotoxicity in six patients (11.3%) in the PT group and no patients in the biapenem group (*P* = 0.005) [22]. This study included only elderly patients and PT was compared with biapenem only, which is neither approved in the United States nor in Europe, which limits its external validity to other β-lactams. Additionally, the authors did not discuss the use of other nephrotoxic agents or antibiotics that might have contributed to the increased incidence of AKI, nor did they state how they defined nephrotoxicity. Finally, the baseline characteristics of patients did not include baseline kidney function, history of chronic kidney disease, or admission to ICU.

Another study by Jensen et al. evaluated the incidence of kidney failure with broad-spectrum antibiotics, finding that treatment with PT lasting longer than three days was associated with more incidence of estimated glomerular filtration rate of less than 60 ml/min/1.73 m^2^ on day 7 compared to shorter duration of use [17]. Jensen et al. also observed an increase in the rate of renal recovery after the discontinuation of PT. This study examined only the effect of PT on the recovery of renal function but did not directly examine the effect of PT on the incidence of AKI. Also, patients on vancomycin were not excluded and the analysis was not adjusted for the use of vancomycin or aminoglycosides, which might have led to a slower recovery if they were used concomitantly with PT. Finally, this finding about PT was detected from a secondary analysis which makes it exploratory and needs to be confirmed in further studies.

Recently, Hall et al. found that PT is not an independent risk factor for AKI among patients with Gram-negative bacteremia. However, only one-fifth of the patients were admitted to the ICU which limits the generalizability of the study conclusion to critically ill patients [23]. Another smaller study conducted in Japan showed that PT monotherapy is associated with a higher incidence of AKI compared to cefepime, among critically ill adult patients [19]. In this latter study, only one patient in the cefepime group had AKI and none of the patients in both groups required renal replacement therapy. These findings may reflect the low severity index of the studied cohort, particularly that critical illness severity scores, e.g., APACHE or Sequential Organ Failure Assessment (SOFA) were not reported. This limits the generalizability of these findings to other critically ill cohorts who are generally at a higher risk of AKI. Lastly, a study by Joyce et al. found that PT is an independent risk factor for AKI among critically ill children, compared to cefepime [18]. However, this study included only pediatric patients and did not demonstrate the increased risk of AKI with the combination of PT plus vancomycin which was previously reported among adults. Thus, it is not known whether age plays a role in determining this association.

In the present study, we aimed to assess the renal safety of PT, without vancomycin, in comparison to other commonly used β-lactams among critically ill patients so we excluded patients who received concomitant vancomycin. To avoid the limitations of previous studies, the present study included only critically ill patients, was not restricted to elderly patients, and was adjusted for the concomitant use of nephrotoxic medications in the analysis.

Major surgery is an independent risk factor of AKI [24]. In our study, the PT group had more postoperative patients than the control group, which may reflect a higher risk of AKI among patients in the PT group. However, we repeated our primary analysis and included postoperative status as well as other baseline imbalances in the regression model and did not find a significant difference between the two groups in the odds of AKI.

We found that the length of hospital and ICU stays were longer in the control group. This finding might reflect the pattern of use of these antimicrobials in our facility rather than being an outcome of therapy since we could not calculate the length of stay from the start of therapy, but rather, we analyzed the total length of stay, including the duration before the start of therapy. In our facility, PT is used as the first-line antibiotic when coverage for pseudomonas is indicated, so it is used early in the hospital course. On the other hand, cefepime and meropenem are used mostly for patients who have previous microbiological data showing a pathogen sensitive to either one of them, which is more common among patients with a prolonged hospital stay.

In the present study, the need for renal replacement therapy was higher in the control group (5.6%) than in the PT group (2.2%; *P* = 0.03). This finding might have been caused by the difference in pre-treatment renal function (Table 1). Although we adjusted the analysis of AKI for potential confounders, the number of events in the secondary outcomes, including the need for renal replacement therapy, was not enough to adjust for all potential confounders. Thus, this finding needs to be explored in larger studies.

Due to the structural similarity between teicoplanin and vancomycin and the potential of teicoplanin to cause AKI, we conducted an additional analysis that excluded patients who received teicoplanin concomitantly and found a significantly higher incidence of AKI among patients treated with PT, compared to the control group. This finding, along with the shorter time to AKI among PT group, might indicate a modest association between the use of PT and AKI and warrants larger studies to investigate the renal safety of PT. We suggest that future studies use survival analysis, which might be superior for exploring the difference in the time to AKI.

Limitations of this study include the retrospective design, the relatively small sample size, and the imbalanced number of patients in the study groups (507 patients in the PT group and 162 in the control group). However, this distribution reflects the local pattern of antimicrobial use in our facility, as mentioned earlier. The patients included in this study had low median APACHE II scores at the baseline. However, these scores correlate with the observed mortality. Although most of the baseline characteristics were balanced or adjusted for in the final analysis, there might be some unobserved confounding factors that impacted the results. For example, the source of infection could not be identified from the admission notes for one-third of patients, which precluded its inclusion in the endpoint analysis. Furthermore, we did not assess urine outputs, which may have affected the rate of AKI, especially among elderly patients and patients with low body mass.

## Conclusion

In this study, piperacillin/tazobactam was not associated with a higher risk of AKI compared to cefepime or meropenem among adult critically ill patients. Larger studies may be required to confirm this finding. Additionally, more research is needed to explore the mechanism by which the combination of piperacillin/tazobactam and vancomycin increases the risk of acute kidney injury.

## Electronic supplementary material

Below is the link to the electronic supplementary material.

Supplementary file1 (DOCX 17 kb)
