# Supplementary material for: Evaluation of the risk of acute kidney injury with the use of piperacillin/tazobactam among adult critically ill patients
Source: Infection. 2020 Jul 22;48(5):741–7. doi: 10.1007/s15010-020-01480-x (PMC7518983; doi:10.1007/s15010-020-01480-x)
Supplement: Supplementary file 1 — Supplementary file1 (DOCX 17 kb) [file 15010_2020_1480_MOESM1_ESM.docx]

# Supplementary appendix

**Table 1: Identified sources of infections:**

|  | PT  (n=507) | Control  (n=162) | All patients  (n=669) |
| --- | --- | --- | --- |
| Abdomen | 85 (16.8) | 23 (14.2) | 108 (16.1) |
| Central nervous system | 11 (2.2) | 9 (5.6) | 20 (3) |
| Central line-related | 1 (0.2) | 2 (1.2) | 3 (0.4) |
| Respiratory tract | 173 (34.1) | 37 (22.8) | 210 (31.4) |
| Skin/soft tissue/bone | 41 (8.1) | 7 (4.3) | 48 (7.2) |
| Urinary tract | 25 (4.9) | 26 (16) | 51 (7.6) |
| Unknown/other | 171 (33.7) | 58 (35.8) | 229 (34.2) |

PT: Piperacillin-tazobactam
